# Electrically induced hemodynamic enhancement via sock-integrated electrodes is more comfortable and efficient at 1 hz as compared to 36 hz

**DOI:** 10.1038/s41598-025-97431-3

**Published:** 2025-04-15

**Authors:** Robin Juthberg, Johanna Flodin, Nelida Aliaga, Li Guo, Saul Rodriguez, Nils-Krister Persson, Paul Wilhelm Ackermann

**Affiliations:** 1 Department of Molecular Medicine and Surgery, Karolinska Institutet, Stockholm, Sweden; 2 Smart Textiles and Polymeric E-textiles, Swedish School of Textiles, University of Borås, Borås, Sweden; 3 School of Electrical Engineering and Computer Science, KTH Royal Institute of Technology, Stockholm, Sweden; 4 Department of Trauma, Acute Surgery and Orthopaedics, Karolinska University Hospital, Stockholm, Sweden

**Keywords:** Electric stimulation therapy, Textile electrodes, Motor point, NMES, Hemodynamics, Pain, Thromboembolism, Rehabilitation, Preclinical research, Preventive medicine, Orthopaedics

## Abstract

This study evaluated the hemodynamic effects, discomfort, and energy efficiency of low-intensity neuromuscular electrical stimulation (LI-NMES) of the calf delivered via sock-integrated transverse textile electrodes (TTE) at different frequencies and plateau times. Fifteen healthy participants underwent NMES stimulation through 3 × 3 cm TTE with ten combinations of frequency (1–36 Hz) and plateau times (0.5–7 s). NMES was increased until plantar flexion occurred, at which point ultrasound-measurements were made of popliteal peak venous velocity (PVV), time-averaged mean velocity (TAMV), average duration of blood flow pulse (ADBP) and ejection volume (EV). Discomfort (NRS, 0–10), current amplitude, and energy consumption were recorded. Median values were analyzed with significance set at *p* < 0.05. Both 1 Hz and 36 Hz C-LI-NMES significantly improved PVV and TAMV (*p* ≤ 0.008). EV increased significantly for plateau times of 1.5, 5.0, and 7.0 s (*p* < 0.05). Compared to 36 Hz, 1 Hz showed significantly lower discomfort (NRS: 0.4 vs. 1.6) and energy consumption (0.4 vs. 31.3 mJ, both *p* ≤ 0.01) but required higher current amplitude (33.2 vs. 23.3 mA, *p* < 0.01) to reach plantar flexion. The study concludes that both 1 Hz and 36 Hz frequency improve venous hemodynamics, but 1 Hz stimulation minimizes discomfort and energy use while maintaining effectiveness.

**Trial registration**: Retrospectively registered with Clinical Trials, trial ID: NCT06082297.

## Introduction

Venous thromboembolism (VTE), which encompasses deep vein thrombosis (DVT) and pulmonary embolism, remains a critical clinical challenge—particularly among patients experiencing prolonged immobilization^1,2^. Conventional active mechanical prophylaxis, such as intermittent pneumatic compression (IPC) devices, has demonstrated efficacy in promoting venous return; however, their impact is often limited by issues of patient compliance and cumbersome device designs^3^. Static graduated compression stockings, on the other hand, which lack active intermittent compression, have demonstrated no additional benefit in preventing VTE and VTE-related mortality among surgical inpatients in a recent systematic review^4,5^. Other recent systematic reviews and guidelines underscore the urgent need for improved strategies to reduce the risk of VTE in surgical and non-surgical patients^6–9^. Given the widespread burden of VTE, particularly among populations with limited mobility, the potential for NMES as a scalable prophylactic intervention warrants further investigation.

Neuromuscular electrical stimulation (NMES) has emerged as an attractive alternative because it can engage the body’s own natural calf muscle pump, thereby enhancing venous return and reducing stasis, with higher patient mobility and less complexity as compared to IPC^10^. Low-intensity NMES (LI-NMES) protocols have shown that clinically relevant hemodynamic improvements can be achieved with reduced discomfort and lower energy requirements^11–14^. Notably, Ravikumar et al. recently conducted a randomized controlled trial in non-operative venous disease and found that NMES significantly improved both clinical and symptomatic status, suggesting that NMES may play a role in reducing venous stasis^15^. Similarly, a study by Calbiyik and Yilmaz reported that NMES can increase femoral and lower limb venous blood flow in post-surgical settings^16^. Zhao et al. demonstrated improved recovery after total hip replacement surgery using NMES, while Ma, Wang, and Cui explored the positive effects of IPC combined with NMES on blood flow and DVT prevention after hip arthroplasty^17,18^. These recent findings, along with recent consensus reports on the risk assessment for VTE, emphasize the clinical potential of NMES as a non-pharmacological intervention for thromboprophylaxis^8,9^.

The epidemiology of DVT underscores the urgent need for accessible prophylactic interventions. In the United States, the annual incidence of DVT is estimated at approximately 1 to 2 cases per 1,000 individuals, with higher rates observed among older adults, post-operative patients, and those with limited mobility^19–21^. Globally, millions of physically inactive individuals are at risk for DVT—a condition that not only contributes to significant morbidity and mortality but also imposes considerable healthcare costs^22,23^. Portable NMES devices integrated into everyday wear offer a promising solution for at-home VTE prophylaxis by providing continuous, non-invasive treatment that can be seamlessly incorporated into daily routines. By decreasing the risk of VTE-related complications, these devices could help reduce hospital readmissions, the need for extended anticoagulation therapy, and the financial burden associated with recurrent VTE events while also improving the quality of life for at-risk populations. Beyond VTE prevention, the versatility of portable LI-NMES systems extends to the management of chronic venous insufficiency and rehabilitation following musculoskeletal injuries, thus broadening their clinical applications^24^.

Parallel to these clinical insights, advancements in wearable technology and smart textiles have significantly transformed how NMES can be administered to patients. For example, Al-Rasheed et al. recently presented the development of industry-scalable, flexible textile electrodes capable of delivering effective and consistent neurostimulation with optimal comfort, that can enable the incorporation of NMES systems into everyday garments^25^. These advancements enhance the possibilities for patients to self-administer NMES therapy without requiring clinical supervision, enabling consistent use in home settings and improving adherence to thromboprophylaxis guidelines. For example, calf-NMES could potentially be integrated into socks to improve everyday accessibility and thereby adherence to thromboprophylactic treatment. However, this would likely require a significant reduction in the size of the batteries and other electrical components of the NMES-device, which may be achieved by reducing the stimulation energy used to induce the blood flow increasing muscle contractions.

Despite these promising developments, comprehensive studies that systematically explore the influence of various NMES parameters on venous hemodynamics remain limited. While traditional high-intensity protocols have dominated past research, recent evidence indicates low-intensity neuromuscular electrical stimulation of the calf (C-LI-NMES) can elicit comparable improvements in venous flow—with added advantages of lower discomfort and better energy efficiency. To investigate this, our research group recently published two feasibility studies investigating a novel sock with integrated transverse textile electrodes (TTE) where the concept of C-LI-NMES was introduced, i.e., using the minimal amount of stimulation energy that can produce a clinically relevant hemodynamic effect^12,13^. The first study suggested that 1–36 Hz (Hz) C-LI-NMES via TTE may both produce ankle plantar flexion, but indicated a lower discomfort and energy consumption when using 1 Hz^11,12^. The second study, which only investigated 36 Hz, suggested that a plantar flexion induced by C-LI-NMES via TTE, regardless of stimulation duration (i.e. plateau time), can significantly improve hemodynamics^13^. However, the relative effects on hemodynamics, comfort and energy efficiency if using 1 Hz vs. 36 Hz combined with different plateau times during C-LI-NMES via TTE have not previously been investigated^12,13^. This is particularly relevant for VTE prophylaxis, where patient adherence to treatment and long-term usability are paramount^26^.

To address these gaps, the present study investigates the effects of C-LI-NMES delivered via sock-integrated textile electrodes at two different frequencies (1 Hz and 36 Hz) and across a range of plateau times (0.5–7 s) on key hemodynamic parameters. By evaluating a spectrum of stimulation settings, our work aims to identify the optimal balance between efficacy, patient comfort, and energy consumption—thereby laying the groundwork for both enhanced clinical applications and innovative at-home DVT prophylaxis solutions.

## Methods

### Participants and study design

This was an exploratory repeated measures design study including 15 healthy participants, i.e. without any known acute or chronic disease. The study investigated the effect of different NMES parameter settings on popliteal vein hemodynamic outcomes as well as discomfort. Inclusion criteria were age ≥ 18 and voluntary participation. Exclusion criteria were pregnancy, pacemaker, ongoing thromboprophylaxis, skin wounds, vascular abnormalities or previous vascular system surgery in the lower limbs. All participants were asked to sign an informed consent and to fill in information about themselves, including sex, age, height, weight, BMI, tobacco use and estimated physical activity (1–6) according to the Grimby/Frändin activity scale^27^ (Table 1).

**Table 1:** Participant characteristics (*n* = 15).

Variable	
Sex, male, n (%)	9 (60)
Age, years, M (IQR)	29 (26–50)
Height, cm, M (IQR)	174 (170–180)
Weight, kg, M (IQR)	75 (62–92)
BMI, M (IQR)	25 (21–29)
Tobacco use, yes, n (%)	4 (27)
Physical activity ^a^, M (IQR)	5 (3–5)

*M* median, *IQR* inter-quartile range, *BMI* body mass index.

^a^Frändin/Grimby activity scale (range 1–6).

## Materials

### Sock-integrated transverse textile electrodes (TTE)

The prototype used in this study is a compression sock featuring a pair of integrated textile electrodes. These electrodes are oriented transversely within the sock and are therefore named Transverse Textile Electrodes (TTE). The prototype sock is identical to the one used and described in depth in our previous studies^12,13^. As a medium to facilitate the electrical conduction, saline solution (0.9% isotonic sodium chloride ion containing solution) was chosen. Furthermore, as compared to commercial liquid gels, the saline solution was better absorbed and distributed within the melamine sponge in contact with the TTE of the sock, allowing for a much better spread of the conductive medium across the entire electrode-skin interface. Additionally, the tested TTE-socks had light compression and also elastic yarn floats over the melamine sponge, which further created local pressure that lightly and evenly pressed the whole TTE against the skin, minimizing the risk of any local hot spots. The TTE were connected to a constant current NMES device (Chattanooga Physio, DJO Nordic, Malmö, Sweden).

### Protocol for measurement of outcomes

The investigated hemodynamic parameters were peak venous velocity (PVV) in centimeters per second (cm/s), time-average mean velocity (TAMV) in cm/s, average duration of blood flow pulses (ADBP) in seconds (s) and ejection volume (EV) in milliliter (ml) in the popliteal vein during ankle plantar flexion. Directly after each hemodynamic measurement, participants were verbally asked to estimate discomfort from 0 to 10 in steps of 1 using a numerical rating scale (NRS), where 0 corresponded to no discomfort and 10 corresponded to the worst imaginable discomfort. For all tests in this study, participants were seated in a regular chair with the knees flexed approximately 45 degrees to allow access for an ultrasound doppler (Philips CX50, 2013, Philips Medical Systems, Andover, MA, USA) measurements of the blood flow of the popliteal vein. All ultrasound measurements were performed by two researchers alternating the role as the primary examiner (i.e. holding the ultrasound probe) for each participant, where the other researcher served as an assistant. For each test, the hemodynamics of the popliteal vein was first examined at rest (baseline), and then again during C-LI-NMES induced plantar flexion. The leg side that was tested and examined with the ultrasound was randomized for each patient. Each round of testing the different NMES settings started by applying NMES with the lowest possible current amplitude (5.5 mA) followed by as small as possible stepwise increases (typically 1 mA), until one of three outcomes occurred; (1) a NMES induced plantar flexion of the ankle became visible to the examiner, (2) the test was aborted by the examiner due to insufficient electrical connection, or (3) the test was aborted by the participant due to discomfort, at which point the NMES test round ended. The procedure did not use fixed time intervals between current level increments; instead, with the consent of the participant, the current was continuously stepped up as soon as it was clear to the examiner that the previous current level had not yet elicited plantar flexion. Typically, one or two stimulation cycles were required on each current level to determine if a plantar flexion could be observed. Each stimulation cycle could consist of ramp-up time 0.5 s, plateau time 0.5/1.5/3/5–7 s, ramp-down time 0.5 s and OFF-time 6 s. Consequently, the entire stimulation round typically lasted between 20 and 60 s before plantar flexion was observed. Among all the 150 tests performed in the 15 participants, only two tests were aborted due to insufficient electrical connection, and this was not considered enough loss of data to exclude those patients´ other data-points from the study. No tests were aborted due to pain. When plantar flexion was observed, measurements of the hemodynamic outcomes, discomfort, current amplitude in milliampere (mA) and energy consumption in millijoule (mJ) were performed. The energy consumption for each stimulation cycle was calculated using the conversion-formula previously published by our research group. Plantar flexion of the ankle induced by the lowest possible NMES intensity was chosen as the threshold of measurement for outcomes since previous studies have shown that it induces clinically relevant increases in popliteal vein blood flow with low associated discomfort^11–13,28^.

**Fig. 1:**
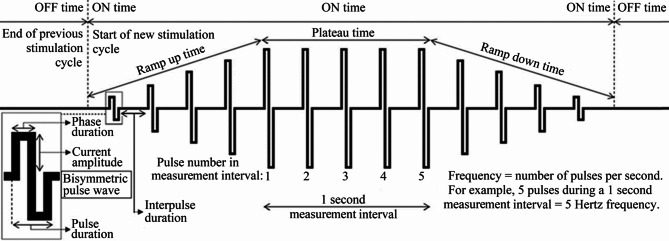
Examples of neuromuscular electrical stimulation parameters that may be adjusted to minimize current requirements and discomfort.

The tested NMES settings used a bisymmetric rectangular wave with stimulation frequencies 1 and 36 Hz combined with each of the plateau times 0.5, 1.5, 3, 5 and 7 s. For all stimulations using 36 Hz, a 0.5 s ramp-up time and 0.5 s ramp-down time was used to minimize discomfort as suggested by^29^. Phase duration was set to 200 µs for all tests and each stimulation cycle was followed by a 6 s OFF-time (Fig. 1).

### Statistics

Descriptive statistics for participant characteristics were presented as medians and inter-quartile ranges (IQR) while inferential statistics were presented with median and IQR. Because of the limited number of participants, and to limit the effect of outliers, the non-parametric Wilcoxon signed rank test was used to calculate differences between outcomes, which were considered significant if *p* **≤** 0.05. All data were analyzed and all boxplots were created using SPSS (version 28 for Windows, Armonk, NY: IBM Corp.).

The study investigated the effect of two different NMES frequencies (1 and 36 Hz) and five different plateau times (0.5, 1.5, 3, 5 and 7 s) on four hemodynamic outcomes (PVV, TAMV, ADBP and EV), discomfort (NRS), current amplitude and energy required to induce ankle plantar flexion, as well as hemodynamic outcomes at baseline. Comparisons between all frequency/plateau time-combinations, as well as comparisons to baseline, would render a total of 55 possible related samples comparisons for each outcome variable, which solely based on the larger number of comparisons would increase the risk of some comparisons erroneously indicating significant differences.

To control for such statistical errors, the research design was specifically planned ahead to only include comparisons between frequencies (one comparison), between frequencies and baseline (two comparisons), between plateau times including baseline (15 comparisons), and for the special case of energy, between the 1 Hz single pulse and each of the five different plateau times combined with the 36 Hz frequency (15 comparisons). This was achieved by averaging out the outcome scores for the parameter not in focus to which non-parametric Wilcoxon signed rank test was applied to determine if there were any statistical differences in the outcomes. Outlier data points were considered to be the result of natural variations and were therefore included in the statistical analysis. Intervention-related changes in hemodynamic outcomes were reported as percentages of change and effect sizes were statistically calculated using rank-biserial correlation (rB) for Wilcoxon signed-rank tests.

## Results

### Effect of C-LI-NMES frequency on hemodynamic parameters when applied via TTE

**Fig. 2:**
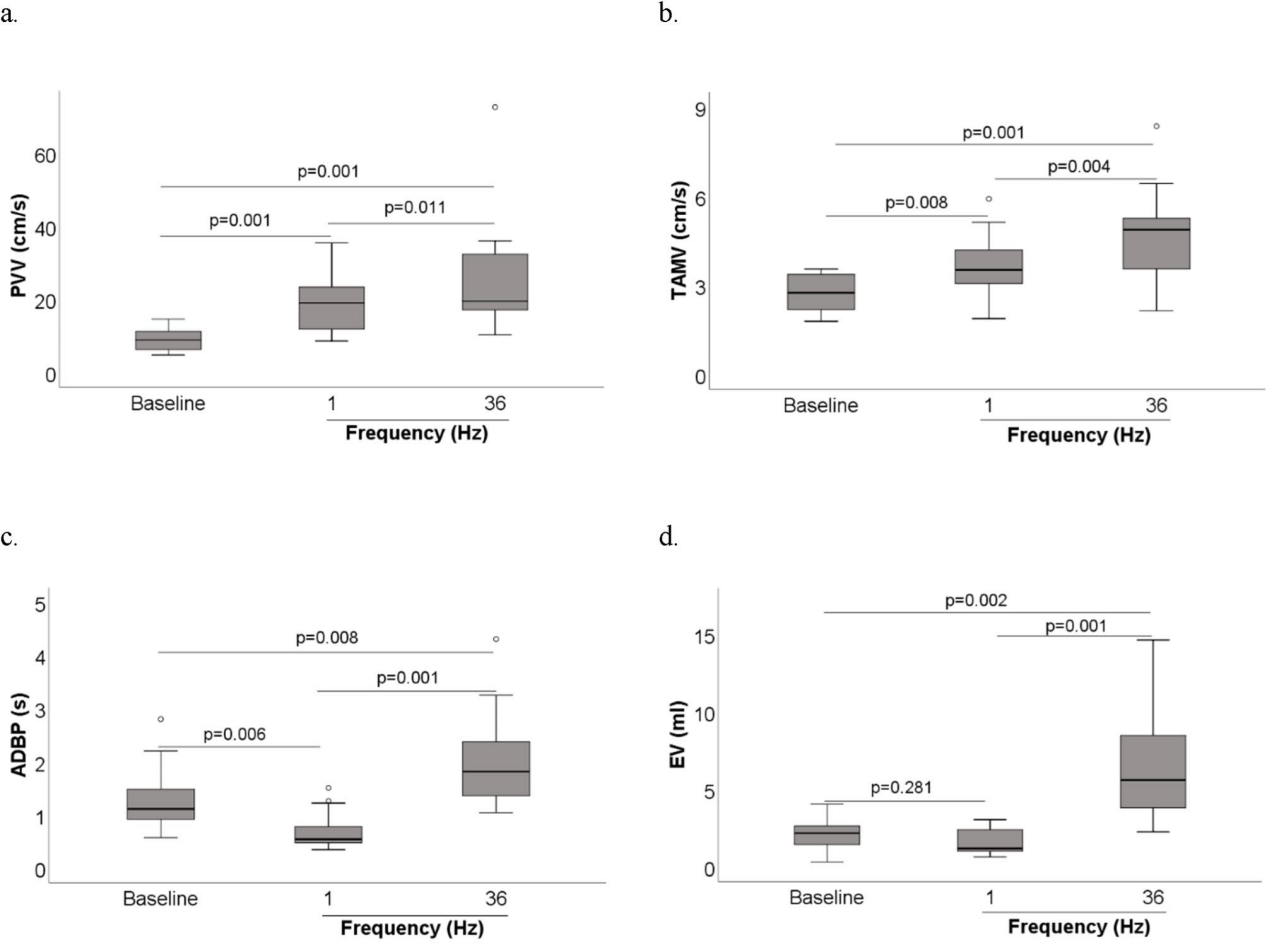
(**a**–**d**) Comparisons of hemodynamic parameters at baseline, 1 Hz and 36 Hz. Line between boxplots indicates statistically significant difference at *p* < 0.05. Circle indicates outlier. Abbreviations: PVV = peak venous velocity, TAMV = time-average mean velocity, ADBP = average duration of blood flow pulse, EV = ejection volume, Hz = Hertz.

Ankle plantar flexion induced with C-LI-NMES through a TTE sock using a 36 Hz frequency, produced significant increases compared to baseline in all hemodynamic parameters studied, i.e. PVV, TAMV, ADBP and EV, in the popliteal vein (all *p* ≤ 0.008). When using 1 Hz, significant increases in popliteal vein hemodynamics relative to baseline were seen for PVV and TAMV (both *p* ≤ 0.008), but not for ADBP or EV (Fig. 2).

### Peak venous velocity (PVV) in the popliteal vein

The median (IQR) PVV at baseline, 1 Hz and 36 Hz was 8.9 (6.2–12), 19 (12–24) and 20 (17–33) cm/s, respectively. Compared to baseline, NMES with both 1 Hz and 36 Hz resulted in significant increases in PVV by 2.1-fold and 2.2-fold, respectively (both *p* ≤ 0.001 and rB = 1.000). The median PVV at 36 Hz was also significantly higher than at 1 Hz (*p* = 0.011, rB = 0.875) (Fig. 2a).

### Time-averaged mean velocity (TAMV)

The highest median TAMV of 4.9 (3.5–5.4) cm/s was produced at 36 Hz, corresponding to a 75% significant increase over the baseline TAMV of 2.8 (2.2–3.4) cm/s (*p* = 0.001, rB = 1.000) and a 40% significant increase of TAMV as compared to that produced with 1 Hz, which was 3.5 (2.9–4.2) cm/s (*p* = 0.004, rB = 0.925). The 1 Hz C-LI-NMES stimulation also produced significantly higher TAMV compared to baseline (*p* = 0.008, rB = 0.896) (Fig. 2b).

### Average duration of blood flow pulse (ADBP)

C-LI-NMES with 1 Hz produced the shortest ADBP of 0.54 (0.47–0.82) s, which was significantly shorter than both the ADBP at baseline of 1.1 (0.87–1.5) (*p* = 0.006, rB = 0.892) s and the ADBP resulting from 36 Hz which duration was 1.8 (1.2–2.4) (*p* < 0.001, rB = 1.000) s. Compared to baseline, 36 Hz prolonged the ADBP by 60% (*p* = 0.008, rB = 0.892) (Fig. 2c).

### Ejection volume (EV)

The baseline EV of 2.2 (1.3–2.7) ml was not significantly different from the EV produced with 1 Hz C-LI-NMES, which was 1.2 (0.96–2.5) ml. However, the EV produced at 36 Hz of 5.6 (3.7–10) ml was 2.5-fold and 4.7-fold higher as compared to baseline (*p* = 0.002, rB = 0.958) and 1 Hz (*p* < 0.001, rB = 1.000), respectively (Fig. 2d).

### Effect of C-LI-NMES plateau times on hemodynamic parameters when applied via TTE

Among the different hemodynamic parameters, PVV and TAMV exhibited significant increases with the largest effect sizes relative to baseline when increasing the plateau time, whereas ADBP and EV exhibited the most pronounced effects when varying the different plateau times (0.5, 1.5, 3, 5 and 7 s).

### PVV

**Fig. 3:**
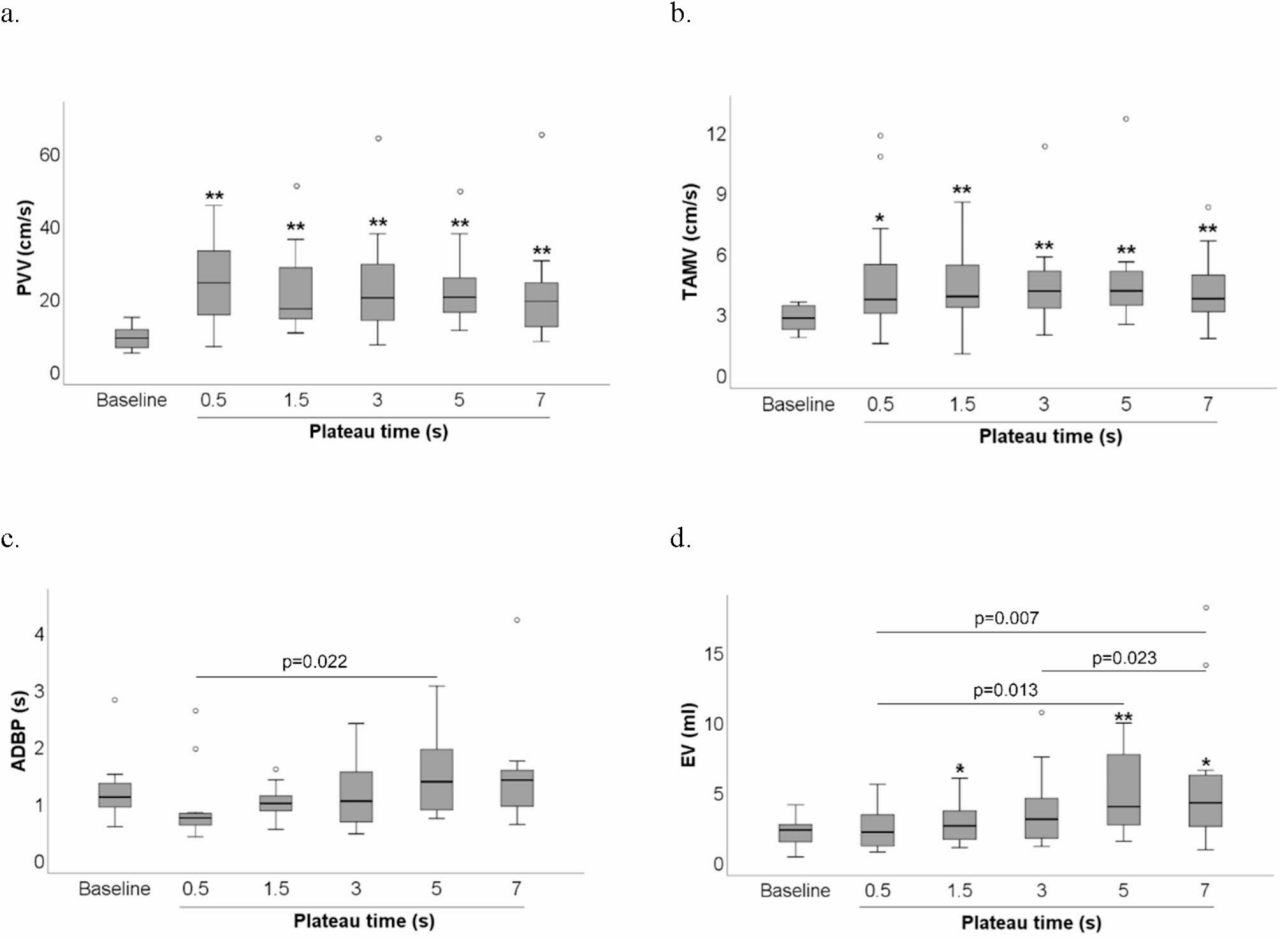
(**a**–**d**) Comparisons of hemodynamic parameters at baseline v different plateau times. Single asterisk indicates statistically significant difference compared to baseline at *p* < 0.05. Double asterisks indicate statistically significant difference compared to baseline at *p* < 0.01. Line between boxplots indicates statistically significant difference at *p* < 0.05. Circle indicates outlier. Abbreviations: PVV = peak venous velocity, TAMV = time-average mean velocity, ADBP = average duration of blood flow pulse, EV = ejection volume, Hz = Hertz.

The highest PVV was seen for plateau time 0.5 s, which had a PVV of 24 (14–33) cm/s, exhibiting a 2.7-fold significant increase vs. the baseline PVV of 8.9 (6.2–12) cm/s (*p* < 0.001, rB = 1.000) (Fig. 3a). No significant differences were found in median PVV between the different plateau times.

### TAMV

When looking at median TAMV, the greatest significant increases from the baseline of 2.8 (2.2–3.4) cm/s was seen for plateau time 3 s which had 4.1 (3.1–5.3) cm/s and 5 s with 4.1 (3.4–5.2) cm/s (both *p* ≤ 0.005, rB = 0.925–0.983) (Fig. 3b). No significant differences were found in median TAMV between the different plateau times (0.5, 1.5, 3, 5 and 7 s).

### ADBP

None of the plateau times resulted in a significant difference in ADBP compared to baseline. However, a 5 s plateau time resulted in a significantly longer median ADBP of 1.5 (0.86–2.0) s as compared to the 0.5 s plateau time of 0.76 (0.62–1.1) s (*p* = 0.022, rB = 0.852) (Fig. 3c).

### EV

Plateau times 1.5, 5 and 7 s resulted in EV of 2.6 (1.8–5.9) ml, 4.4 (2.6–8.2) ml and 4.2 (2.5–8.4) ml respectively, all significantly higher vs. baseline, which was 2.2 (1.3–2.7) ml (all *p* < 0.023, rB = 0.833–0.900) (Fig. 3d). The EV induced by 5 s and 7 s were significantly higher (both *p* < 0.013, rB = 0.876–0.923) compared to the EV induced by a plateau time of 0.5 s, which was 2.5 ml (1.1–3.5) (Fig. 3d). Furthermore, a 7 s vs. a 3 s plateau time also resulted in a significantly higher median EV (*p* = 0.023, rB = 0.857).

### Effect of C-LI-NMES frequency and plateau times on discomfort when applied via TTE

**Fig. 4:**
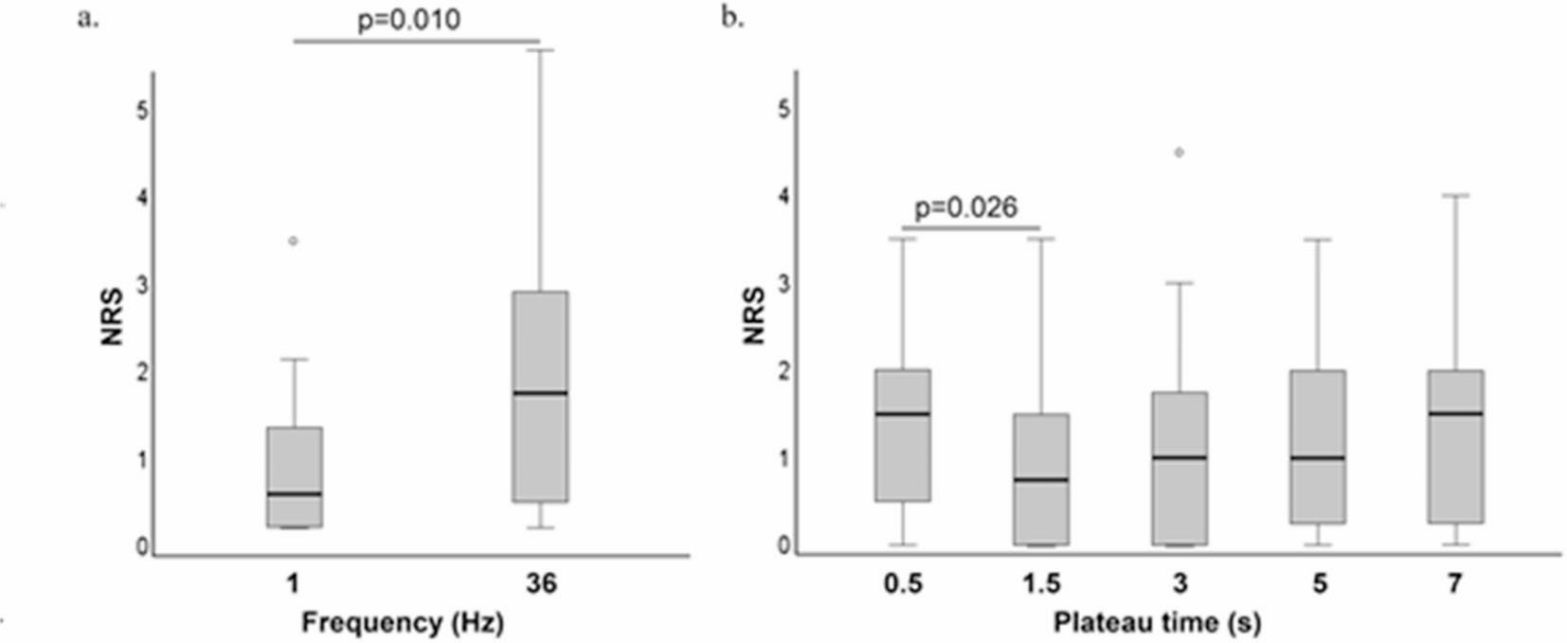
(**a**,**b**) Comparisons of discomfort (NRS) for different frequencies and plateau times. Line between boxplots indicates statistically significant difference at *p*<0.05. Circle indicates outlier. Abbreviations: NRS = numerical rating scale, Hz = Hertz.

When evaluating discomfort, the median NRS at 1 Hz of 0.4 (0–1.2) was significantly lower compared to the NRS 1.6 (0.2–3.2) induced with C-LI-NMES using 36 Hz (*p* = 0.010) (Fig. 4a). The only significant difference regarding NRS between plateau times was that 0.5 s plateau time exhibited a significantly higher median NRS of 1.5 (0.5–2.5) as compared to 1.5 s plateau time with a median NRS of 1.0 (0–2.5) (*p* = 0.026) (Fig. 4b).

### Effect of C-LI-NMES frequency and plateau times on required current amplitude when applied via TTE

**Fig. 5:**
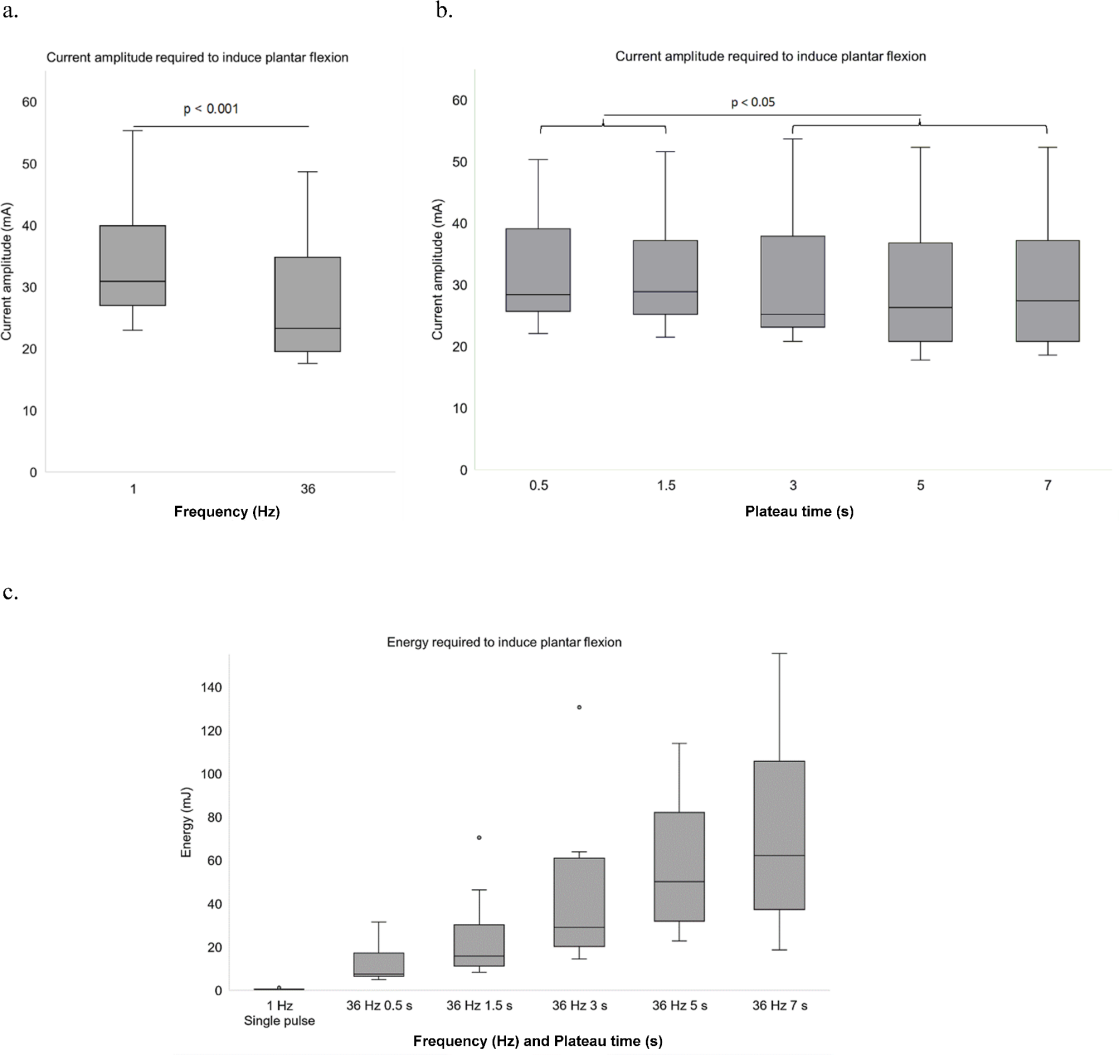
(**a**–**c**) Comparisons of required current amplitude and energy for different frequencies and plateau times. (**a**) line between boxplots indicates significant difference at *p* < 0.001. (**b**) the line overlaying the two brackets signifies that there is a statistical difference in mA for each condition under the left bracket when compared to the mA value for any of the conditions under the right bracket. (**c**) in addition to the outliers seen in the figure, there are two outliers outside of the scale, one for 36 Hz 5 s at 182.5 mJ and one for 36 Hz 7 s at 248.8 mJ. All outliers represent data points from one patient. All comparisons are significant at *p* < 0.001. mA = milliampere, Hz = Hertz, mJ = millijoule.

Significantly more current was required to induce plantar flexion with 1 Hz at 33.2 (25.4–55.2) mA, as compared to 36 Hz which required 23.3 (19.5–38.4) mA (*p* < 0.001) (Fig. 5a). Each of the plateau times 3, 5 and 7 s required lower current to induce plantar flexion, with mA-medians in the range 25.2–27.4 (20.8–37.9), as compared to each of the plateau times 0.5 and 1.5 s, which required in median 28.4 (25.7–39.1) and 28.9 (25.7–39.1) mA, respectively (all *p* < 0.05) (Fig. 5b).

### Effect of C-LI-NMES frequency and plateau times on energy-requirements when applied via TTE

Plantar flexion induced by a stimulation cycle using a single 1 Hz pulse required a median energy of 0.4 (0.3–0.6) mJ, significantly less than each stimulation-cycle using 36 Hz together with any of the plateau times with mJ medians ranging from 7.5 to 62.5 mJ with increasing plateau time (all *p* < 0.001). At 36 Hz, each increase of plateau time required significantly higher mJ compared to each of the shorter plateau times (all *p* < 0.001) (Fig. 5c). The median energy required to induce plantar flexion when including all plateau times during 36 Hz C-LI-NMES was 31.3 (22.1–61.1) mJ, which is approximately 78 times more energy compared to a single 1 Hz pulse.

## Discussion

To the best of our knowledge, this is the first exploratory study examining the effect of frequencies 1 and 36 Hz, and plateau times 0.5, 1.5, 3, 5 and 7 s, on hemodynamic parameters PVV, TAMV, ADBP and EV as well as discomfort, current amplitude and energy consumption when inducing a plantar flexion of the ankle with C-LI-NMES administered via TTE integrated into a sock. The findings showed that C-LI-NMES at both 1 Hz and 36 Hz significantly increased PVV and TAMV in the popliteal vein as compared to baseline. However, 36 Hz frequency induced significantly higher hemodynamic output in all parameters as compared to 1 Hz. Conversely, 1 Hz was associated with significantly lower discomfort and energy consumption, which may have important implications for patient adherence to long-term NMES therapy. All plateau times produced significantly higher PVV and TAMV compared to baseline, while 1.5 s plateau time induced significantly less discomfort compared to 0.5 s.

### Main findings

The main finding of this study was that when inducing a plantar flexion of the ankle with C-LI-NMES via TTE, with either 1–36 Hz and any of the five tested plateau times, popliteal vein PVV and TAMV increased significantly compared to baseline. Since venous stasis according to Virchow’s triad is a key risk factor for VTE, these findings suggest that NMES via TTE socks could be a viable thromboprophylactic intervention. Due to a lack of studies investigating the correlation between different hemodynamic parameters and the incidence of VTE, there is no generally accepted consensus regarding which hemodynamic parameter is best suited to estimate the risk of VTE. Broderick et al. suggested that an increased EV coincides with less blood left in the veins and thereby a decreased stasis, which potentially could decrease the risk of DVT^29^. The cause of a venous thrombus in the deep veins may however also be caused by slow venous flow, which may suggest that hemodynamic parameters such as PVV and TAMV would be better predictors of DVT incidence^29^. Currently this seems to be the predominant hypothesis, as most recent hemodynamic studies performed in the context of VTE prophylaxis primarily investigate PVV, regardless of method used to reduce stasis^30–36^.

Our recent studies demonstrated that plantar flexion induced by 36 Hz C-LI-NMES via TTE, regardless of plateau time, can produce significant increases in PVV in the popliteal vein. The studies also demonstrated that the stimulation cycle using a 1 Hz single pulse requires about 25 times less energy compared to a stimulation cycle using 36 Hz with a 1.5 s plateau time to produce the same level of plantar flexion^12,13^. In this study we corroborated the conception that 1 Hz is more energy efficient than 36 Hz and also expanded our investigation to include a wider spectrum of hemodynamic outcomes while also testing new parameter settings that may be relevant to apply in an everyday use setting.

### Therapeutic implications of 1 hz vs. 36 Hz NMES

A key observation was that 36 Hz produced a nearly fivefold increase in EV compared to 1 Hz, whereas PVV and TAMV exhibited smaller relative differences between the two frequencies. Another important observation was that 36 Hz was significantly less comfortable and energy-efficient when inducing the plantar flexion. Our main theory for why 1 Hz caused less discomfort is that there is a fine line between a controlled tetanized muscle contraction, and a painful muscle cramp, where the latter can be induced with the summation of 36 pulses per second, but not by a single pulse. This suggests that while 36 Hz leads to a stronger venous emptying effect, the added benefit in reducing venous stasis may not be proportional to the increase in discomfort and energy consumption. Given that patient compliance is essential for the effectiveness of NMES-based VTE prevention, the trade-off between hemodynamic efficiency and tolerability must therefore be carefully considered^12,29^.

Conversely, the lower discomfort induced with 1 Hz, combined with its significant PVV and TAMV improvements over baseline, suggests that it may offer a more practical and tolerable approach for long-term adherence to thromboprophylactic treatment, especially in home-based or post-operative settings. This is important because devices aiming to facilitate continuous venous return are highly dependent on patient compliance. For example, for VTE prevention of postoperatively immobilized patients, efforts should be made to achieve 18 h of daily compliance according to the international guidelines of American College of Chest Physicians^26^. While the results of the present study were obtained under controlled conditions, they provide a strong proof-of‐concept for the potential clinical application of sock-integrated NMES to optimize DVT prophylaxis. Future clinical trials should evaluate whether the trade-off between hemodynamic efficiency at 36 Hz and comfort-driven compliance at 1 Hz translates into differences in real-world VTE prevention outcomes.

In terms of muscle contraction effectiveness during plantar flexion, 36 Hz resulted in tetanic contractions, which are more sustained and produce greater venous compression, leading to higher EV. In contrast, 1 Hz produced individual muscle twitches which resulted in lower EV, but sufficient PVV and TAMV increases to still suggest a clinically relevant effect for VTE prophylaxis. The discovery that 36 Hz was more hemodynamically effective than 1 Hz is not unexpected, although novel when it comes to textile integrated electrodes. Earlier studies, using standard gel electrodes, confirm the findings that higher frequencies result in a more effective muscle contraction, which leads to higher compression of blood vessels and presumably explain the improved hemodynamics^10,29,37,38^. The most likely explanation why 36 Hz stimulation required lower current amplitudes to achieve plantar flexion, is the phenomenon of tetanization^37^. At a higher frequency like 36 Hz, each NMES pulse adds to the residual effect of the previous pulse, leading to a sustained contraction through temporal summation. This tetanization means that less current is needed per pulse to achieve the same degree of muscle contraction compared to 1 Hz stimulation, where individual pulses are isolated and the muscle fully relaxes between them, necessitating higher current to induce plantar flexion. One possible downside of using a higher frequency could be the increased risk of muscle fatigue, which might have potential long-term implications when choosing between 1 vs. 36 Hz NMES^39^.

### Relevance of plateau time in NMES optimization

Our study also examined the impact of plateau time on hemodynamic outcomes. Unlike frequency, plateau time did not significantly influence PVV or TAMV, suggesting that the duration of muscle contraction may be less important than the stimulation frequency in optimizing hemodynamic benefits. However, longer plateau times were generally associated with significant increases in EV, indicating that longer plateau times may be more efficient in promoting venous return. Longer plateau times were also associated with increased ADBP, but only the difference between 0.5 s and 5.0 s reached statistical significance. Reported discomfort showed an interesting pattern where the shortest plateau time of 0.5 s caused significantly more discomfort vs. 1.5 s which had the lowest reported discomfort, but where discomfort thereafter increased with longer plateau times. A possible explanation for this could be that the shorter plateau time of only 0.5 s may have been perceived as more of an “electrical shock” to the participants, while the longer 1.5 s plateau time might have been perceived as more of a natural controlled muscle contraction. The observed increase in discomfort with increasing plateau times beyond 1.5 s, may suggest that participants experienced a gradual transition from a perceived natural muscle contraction to more of an uncomfortable cramping feeling. Interestingly, the most comfortable plateau time of 1.5 s, was not significantly worse than any other plateau time regarding any one of the four hemodynamic measurements.

From a clinical perspective, the limited number of significant increases in hemodynamic parameters when increasing the plateau time, suggests that plateau times longer than 1.5 s may not provide substantial additional hemodynamic benefits while potentially increasing discomfort or muscle fatigue. Thus, a shorter plateau time of 1.5 s may be preferable, as it balances tolerability and effectiveness. Furthermore, shorter contractions could reduce the risk of muscle fatigue, making NMES more sustainable for prolonged use.

### Energy efficiency and practicality of 1 hz vs. 36 Hz NMES

Additional factors beyond hemodynamics and comfort to consider when stimulating during longer time periods using 1 Hz and 36 Hz include energy consumption and muscle fatigue. Although not investigated in this study, most previous studies on NMES induced muscle fatigue indicate that lower stimulation frequencies are associated with less muscle fatigue^39,40^ Specifically for lower intensity NMES, Doucet et al. showed that a lower stimulation frequency was more effective in reducing muscle fatigue^41^.

Regarding energy consumption, the present study showed that the induction of a plantar flexion using a single 1 Hz C-LI-NMES pulse instead of e.g. 36 Hz NMES during a 7 s plateau time, could reduce the energy consumption by approximately 99.6% per stimulation cycle, while still being able to induce a significant increase in PVV over baseline. To optimize user compliance, reducing energy consumption is crucial for minimizing battery size and the overall device size, making it small enough to be a wearable that naturally integrates into everyday life for long-time use. However, it should be mentioned that the energy efficiency calculations in this study were based solely on the stimulation settings used in the tested device. We did not have the equipment required to measure the overall energy consumption comprehensively. With that said, it is possible that the energy consumption from only the actual electrical stimulation is negligible in relation to the overall energy consumption, but we do not have definitive data on this. Regardless, it is reasonable to assume that a device inducing a single pulse per second would consume less energy on data processing and Bluetooth low energy communication as compared to a device inducing 36 or 50 pulses per second. The assumed lower energy consumption and smaller device size with 1 Hz stimulation could be achieved by using simpler, smaller and fewer components as compared to what would be required in a 50 Hz stimulator device. Thus, further research and development to reduce energy consumption and battery size is indicated to create fully mobile NMES solutions for long-term VTE-preventive use^12^.

Previous studies using high intensity NMES have shown that induction of plantar flexion of the ankle significantly increases PVV over baseline^13,14,28,29^, to a similar degree as IPC devices that are clinically used to prevent VTE in immobilized patients^3,5,13,14,42,43^. However, this is the first study to demonstrate that relevant increases of hemodynamic parameters also can be achieved with both 1 Hz and 36 Hz frequency, when using C-LI-NMES via sock-integrated TTE. Furthermore, this study demonstrated that clinically relevant hemodynamic effects can be achieved with minimal energy consumption and discomfort if stimulating with 1 Hz C-LI-NMES, which may be key factors to reach high adherence to the treatment.

### Strengths and limitations

A within-subject study design was used in our study, in which each participant was exposed to all settings to serve as their own control. The strength of the chosen study design is that it describes the relationship between cause and effect. This is suitable for the purpose of this study and was appropriate based on the small study sample. Since each participant was their own control, the effect of confounders based on the differences in the participants was minimized. Another strength of this study is that the tests were performed according to a protocol regarding position of the participant, setting order and placement of electrodes which made the interventions as similar as possible. A possible limitation of this study is that the generalizability presumably is affected both by participant characteristics and the low number of participants. In future studies more participants and patients with increased risk of DVT should be investigated as well as possible effects of patient characteristics should be explored. A limitation regarding the TTE sock prototype that may affect patient adherence to treatment is that it requires intermittent addition of saline solution every 30 min to keep the melamine sponges moist for good electrical conductivity. Thus, this is an issue that remains to be solved before the TTE sock can be considered fully optimized for patient compliance.

## Conclusions

Low-intensity neuromuscular electrical stimulation of the calf (C-LI-NMES) using sock-integrated, transversely placed textile electrodes (TTE) significantly increases peak venous velocity (PVV) and time-averaged mean velocity (TAMV) in the popliteal vein compared to baseline blood flow at both 1 Hz and 36 Hz, regardless of plateau time (0.5, 1.5, 3, 5, and 7 s). While the more energy-efficient 1 Hz setting was effective, 36 Hz stimulation produced significantly greater hemodynamic improvements but also caused significantly more discomfort. The optimal plateau time of 1.5 s resulted in the lowest perceived discomfort while maintaining similar hemodynamic effects relative to all other plateau times.

These findings suggest that C-LI-NMES via TTE-integrated socks may be suitable for long-term use, offering a promising approach to improving compliance and enhancing the effectiveness of NMES treatments for VTE prevention.

## Data Availability

The data supporting the findings of this study are available from the corresponding author upon reasonable request.
